# Pharmacological Targeting of NRF2 Represents a Promising Therapeutic Approach for Pyroptosis-Related Non-Alcoholic Fatty Liver Disease

**DOI:** 10.2174/0109298673415631251130151116

**Published:** 2026-06-29

**Authors:** Yinggang Zhang, Pengfei Liu, Ying Guo, Kailing Hu, Xinyan Li, Rongrong Liu, Yaping Zhao, Gang Wang

**Affiliations:** 1 Department of Critical Care Medicine, National & Local Joint Engineering Research Center of Biodiagnosis and Biotherapy, The Second Affiliated Hospital of Xi’an Jiaotong University, Xi’an, China;; 2 International Joint Research Center on Cell Stress and Disease Diagnosis and Therapy, National & Local Joint Engineering Research Center of Biodiagnosis and Biotherapy, The Second Affiliated Hospital of Xi’an Jiaotong University, Xi’an, China;; 3 Key Laboratory of Environment and Genes Related to Diseases, Ministry of Education of China, Xi'an Jiaotong University, Xi'an, China;; 4 Key Laboratory of Surgical Critical Care and Life Support, Ministry of Education of China, Xi'an Jiaotong University, Xi'an, China

**Keywords:** NRF2, pyroptosis, NAFLD, NASH, reactive oxygen species, mitochondria, lipotoxicity

## Abstract

Non-alcoholic fatty liver disease (NAFLD) is a clinicopathological condition characterized by the accumulation of fat within hepatocytes in the absence of excessive alcohol consumption or other identifiable causes of liver injury. As a disease involving complex pathogenic mechanisms, NAFLD has become the most prevalent chronic liver disease and may progress to more severe conditions. Pyroptosis is a pro-inflammatory form of programmed cell death that is distinct from classical apoptosis. Accumulating evidence suggests that pyroptosis plays a role in the pathogenesis of NAFLD, contributing to disease progression from simple hepatic steatosis to non-alcoholic steatohepatitis (NASH) and fibrosis. Excessive activation of pyroptosis can exacerbate inflammatory responses, induce cellular damage, disrupt immune homeostasis, and impair liver function. Therefore, elucidating the mechanisms and roles of pyroptosis in NAFLD is crucial for the development of effective therapeutic strategies. As a key transcription factor, nuclear factor erythroid 2-related factor 2 (NRF2) has emerged as a promising therapeutic target. Pharmacological modulation of NRF2 has shown potential in treating diseases characterized by oxidative stress and inflammation. Findings from *in vitro* and animal studies suggest that various compounds that target NRF2 to modulate pyroptosis exhibit notable effects on the initiation and progression of NAFLD. Although most of these agents are still in the early stages of preclinical research, they hold substantial promise for future clinical translation. This review outlines recent advances in pyroptosis-related research in NAFLD and highlights pharmacological targeting of NRF2 as a promising therapeutic approach for pyroptosis-mediated NAFLD.

## 1 INTRODUCTION

NAFLD is characterized by pathological hepatic fat accumulation that exceeds normal physiological thresholds and occurs in the absence of secondary causes of steatosis. As the most prevalent chronic liver disease across various populations, NAFLD encompasses a clinical spectrum ranging from non-alcoholic fatty liver (NAFL) to non-alcoholic steatohepatitis (NASH), with the potential to progress to advanced complications such as cirrhosis, which represents the terminal stage of liver deterioration [1, 2]. Excessive accumulation of fatty acids in hepatocytes represents the primary pathological hallmark of NAFLD. In a subset of patients, the disease may progress to NASH, with the potential development of fibrosis, cirrhosis, hepatic failure, or hepatocellular carcinoma [3]. Factors such as lipotoxicity, oxidative stress, organelle dysfunction, and inflammation can induce hepatocyte death through apoptosis, necrosis, pyroptosis, and ferroptosis, thereby accelerating the progression of NAFLD [4].

Pyroptosis is a distinct form of programmed cell death characterized by cellular swelling and rupture of the plasma membrane. This process results in the release of intracellular contents and the initiation of a strong inflammatory response. Although pyroptosis is physiologically protective through the elimination of intracellular pathogens, excessive activation of this pathway can lead to extensive cell death, tissue damage, organ dysfunction, and ultimately septic shock [5, 6]. Notably, pyroptosis is strongly linked to inflammatory responses.

Inflammation is the body’s essential protective response to injury or infection, aimed at eliminating harmful pathogens and initiating tissue repair. However, chronic or prolonged inflammation can pose significant health risks by causing tissue damage and contributing to metabolic dysregulation. In NAFLD, the liver exhibits infiltration of immune cells, including macrophages and lymphocytes, which secrete pro-inflammatory cytokines and chemokines, thereby amplifying the inflammatory cascade. This persistent inflammatory response represents a key pathological feature of NAFLD, underscoring the need for effective inflammation management to prevent further tissue injury and disease progression [7, 8]. In the inflammatory response associated with NAFLD, multiple signaling pathways are activated. A key mechanism contributing to enhanced hepatic inflammation and cell death is the activation of the NLRP3 inflammasome. This activation is triggered by metabolic stress and the accumulation of fatty acids in hepatocytes. Consequently, inflammatory cytokines are released, further exacerbating liver injury [9]. Controlling inflammation represents a crucial therapeutic strategy for the management of NAFLD. Recent research efforts have focused on developing agents that effectively mitigate hepatic inflammation, including anti-inflammatory drugs and immunomodulatory therapies. Furthermore, lifestyle modifications—such as weight loss, dietary optimization, and increased physical activity—have been shown to significantly reduce liver inflammation and improve the clinical prognosis of NAFLD [10].

NRF2 is recognized as a critical antioxidant transcription factor, and extensive research has demonstrated its capacity to inhibit pyroptosis. This effect is primarily mediated through the activation of antioxidant signaling pathways, resulting in the upregulation of downstream antioxidant genes such as heme oxygenase-1 (HO-1) [11]. Under hypoxic conditions or various forms of physiological stress, intracellular levels of reactive oxygen species (ROS) increase markedly, leading to oxidative damage. Closely associated with oxidative stress, the NRF2 signaling pathway represents one of the most critical cellular defense mechanisms. Under normal physiological conditions, NRF2 binds to Kelch-like ECH-associated protein 1 (Keap1) and remains in an inhibited state. Upon exposure to oxidative stress, NRF2 dissociates from Keap1 and translocates into the nucleus. Once inside the nucleus, NRF2 binds to antioxidant response elements (AREs), thereby activating the expression of numerous antioxidant genes and promoting the synthesis of various antioxidant proteins [12, 13].

In liver tissue, NRF2 is activated by oxidative stress and protects against damage by inducing antioxidant genes such as HO-1 and NQO1 through KEAP1 inhibition. NRF2 also suppresses hepatic inflammation and fibrosis by inhibiting the nuclear factor kappa B (NF-κB) pathway and reducing pro-inflammatory cytokines such as TNF-α and IL-6. In the intestine, NRF2 is activated by microbial products and chemical stimuli, enhancing intestinal barrier integrity through upregulation of tight junction proteins and mucin. It also induces antioxidant enzymes such as SOD and GSH-Px, which reduce ROS and suppress mucosal inflammation by inhibiting NF-κB-mediated cytokine production such as TNF-α and IL-8. The gut-liver axis links both organs, and targeting NRF2 with agents such as polyphenols can simultaneously improve intestinal barrier function and liver oxidative damage, offering a promising approach for treating hepatointestinal comorbidities [14-16].

Furthermore, NRF2 has been identified as a key regulatory factor with protective effects against oxidative damage and inflammatory responses. This article aims to investigate the pathogenesis of NAFLD, elucidate the molecular mechanisms underlying apoptosis, and highlight the NRF2 signaling pathway as a promising therapeutic target for the treatment of NAFLD.

## 2 METHODS

A systematic literature search was performed on PubMed, Embase, and Web of Science, covering the period from each database's inception to April 20, 2025. The search focused on English-language literature related to NRF2, NAFLD, therapeutic strategies for NAFLD, and the pyroptosis pathway. To ensure the scientific validity and reliability of the included studies, only peer-reviewed articles were selected for analysis and synthesis. We believe that investigations into the therapeutic potential of pharmacological agents targeting NRF2-mediated inhibition of pyroptosis hold significant promise for the development of effective treatment strategies for NAFLD.

## 3 THE PATHOGENESIS OF NAFLD

### 3.1 The Insulin Resistance

NAFLD comprises distinct clinical and pathological subtypes. The initial and less severe manifestation, NAFL, is characterized by hepatic lipid accumulation exceeding 5% of hepatocytes, typically in the absence of significant inflammation or hepatocellular injury [17]. NASH is characterized not only by hepatic steatosis but also by necroinflammation and hepatocyte ballooning, which significantly increase the risk of progressive fibrosis, cirrhosis, and ultimately hepatic decompensation or hepatocellular carcinoma if left untreated [18, 19]. The pathogenesis of NAFLD is traditionally explained by the “two-hit” hypothesis. The “first hit” refers to insulin resistance (IR), which leads to hepatic lipid accumulation, whereas the “second hit” encompasses inflammatory responses, oxidative stress, lipid peroxidation, and other related pathological mechanisms. In recent years, this hypothesis has gained increasing recognition in the scientific community [20-22].

The onset and progression of NAFLD are closely associated with visceral obesity. In addition to its role in energy storage, visceral adipose tissue serves as an active endocrine organ that secretes a variety of adipokines, inflammatory cytokines, and metabolic hormones. This altered secretion profile contributes directly to the development of systemic IR and chronic low-grade inflammation. Consequently, increased visceral fat mass is mechanistically linked to hepatic steatosis and commonly coexists with other components of metabolic syndrome [23, 24]. The pathogenesis of NAFLD arises from a complex interaction between genetic predisposition and environmental factors. Importantly, lifestyle-related variables—particularly diets high in calories and fat, obesity, and sedentary behavior—are key modifiable determinants that significantly influence the risk of developing NAFLD [25].

Dysregulated lipid metabolism represents a core pathological mechanism in the progression of NAFLD. Excessive intake of energy-dense, high-fat diets results in an excessive influx of free fatty acids (FFAs) into the liver, thereby promoting the development of hepatic steatosis [26, 27]. IR represents a central pathogenic mechanism underlying this dysregulation of lipid metabolism. IR stimulates lipolysis in adipose tissue, leading to increased systemic FFAs and enhanced hepatic uptake of these lipids. This excessive lipid flux frequently surpasses the liver's metabolic capacity for oxidation and triglyceride (TG) export *via* very-low-density lipoprotein (VLDL), ultimately resulting in intrahepatic triglyceride accumulation [20, 28]. Furthermore, IR reduces glucose uptake and utilization in peripheral tissues while concurrently promoting adipose tissue to release greater amounts of FFAs into the bloodstream. This establishes a self-perpetuating cycle, wherein the increased delivery of FFAs to the liver further aggravates hepatic lipid accumulation [29].

### 3.2 Activation of Inflammatory Factors

Inflammasomes are cytosolic multiprotein complexes that regulate inflammatory responses. These oligomeric structures assemble in response to intracellular danger signals, such as pathogen-associated molecular patterns (PAMPs) or damage-associated molecular patterns (DAMPs), which are released from damaged or stressed cells. Following activation, inflammasomes recruit and activate caspases, thereby initiating robust pro-inflammatory signaling cascades that play a crucial role in the innate immune defense against a wide range of stimuli [30, 31]. Inflammation serves as a central mediator of the inflammatory response, involving pattern recognition receptors (PRRs) that detect conserved molecular structures derived from pathogens. Among the most well-characterized PRR families are the Toll-like receptors (TLRs) and C-type lectin receptors (CLRs). Activation of these PRRs is typically triggered by inflammatory stimuli, leading to the maturation and secretion of key pro-inflammatory cytokines such as interleukin-1β (IL-1β) and IL-18 [32].

Lipid accumulation within hepatocytes and dysfunction of adipocytes in the liver microenvironment induce cellular stress and injury. This pathological damage leads to the release of DAMPs and inflammatory mediators from stressed or apoptotic hepatocytes and adipocytes. These mediators subsequently recruit and activate immune cells, thereby sustaining chronic inflammation, exacerbating hepatocyte injury and necrosis, and facilitating disease progression toward fibrosis [33].

### 3.3 Oxidative Stress

ROS are highly reactive molecules generated during cellular metabolism, including superoxides, hydrogen peroxide, and hydroxyl radicals. Under normal physiological conditions, intracellular ROS levels are maintained at relatively low concentrations and play essential roles in physiological processes such as cell signaling and redox homeostasis. However, exposure to external stimuli or the presence of pathological conditions can lead to a significant elevation in ROS production. When this excessive ROS accumulation surpasses the capacity of cellular antioxidant defense systems, it results in oxidative stress, which may cause cellular damage or even apoptosis [34, 35]. Disruptions in hepatic lipid metabolism lead to ectopic lipid accumulation within hepatocytes. This excessive lipid accumulation subsequently promotes the overproduction of ROS from multiple intracellular sources, particularly dysfunctional mitochondria, the endoplasmic reticulum (ER) under oxidative stress, and activated NADPH oxidase complexes [36].

Recent studies demonstrate that sirtuins (SIRTs) function as potential regulators of lifespan in model organisms. These proteins are critically involved in biological processes essential for kidney development and physiological functions, including mitochondrial metabolism, oxidative stress response, autophagy, DNA repair, and inflammatory regulation. Furthermore, dysregulation of SIRT activity has been implicated in the pathogenesis and progression of various acute and chronic kidney diseases, such as acute kidney injury, diabetic nephropathy, and autoimmune-mediated renal disorders. Accumulating evidence suggests that targeting SIRT signaling pathways may offer therapeutic potential for the prevention and treatment of kidney disease progression [37, 38].

ROS can disrupt insulin signaling pathways and alter the expression and activity of key enzymes involved in lipid metabolism. Supraphysiological levels of ROS cause oxidative damage to cellular components, including plasma membranes, subcellular organelles, and nuclear DNA, ultimately leading to hepatocyte dysfunction and either apoptotic or necrotic cell death [39]. The interplay between lipotoxicity and oxidative stress represents a central mechanism in the pathogenesis of NAFLD. Excessive intracellular free fatty acids serve as potent inducers of oxidative stress, further enhancing ROS production within hepatocytes. Critically, oxidative stress not only mediates direct cellular injury but also actively propagates inflammatory signaling cascades and activates hepatic stellate cells, thereby promoting fibrogenesis [40, 41]. Consequently, oxidative stress is widely acknowledged as a key mechanistic contributor that intensifies hepatocellular injury and critically promotes the progression from simple steatosis to the necroinflammatory changes and fibrotic alterations observed in advanced stages of non-alcoholic fatty liver disease (NAFLD).

### 3.4 Dysbiosis of Intestinal Flora

The concept of the “gut-liver axis” has emerged as a central paradigm in hepatology, providing a framework for understanding the bidirectional communication between intestinal homeostasis and liver health. A key component of this model is the role of gut microbial dysbiosis, which contributes significantly to the initiation and progression of NAFLD through a range of interconnected mechanistic pathways [42]. Experimental and clinical evidence demonstrates that the intestinal microbiota plays a central role in the development of non-alcoholic fatty liver disease (NAFLD) through the following mechanisms: (i) modulation of host energy metabolism and lipid homeostasis; (ii) induction of metabolic endotoxemia due to impaired intestinal barrier integrity; (iii) production of microbiota-derived ethanol; and (iv) alteration of the enterohepatic circulation of bile acids and choline metabolism. Notably, specific bacterial taxa mediate the biotransformation of primary bile acids into secondary bile acids. This microbial metabolic activity disrupts hepatic signaling pathways, thereby promoting hepatic steatosis, inflammation, and fibrotic remodeling in NAFLD [43, 44].

## 4 PYROPTOSIS

Pyroptosis is a lytic and highly inflammatory form of programmed cell death that is distinct from apoptosis. Its key features include cellular swelling, plasma membrane permeabilization (often leading to rupture), and the rapid release of pro-inflammatory cytokines and DAMPs. Importantly, pyroptosis not only leads to the elimination of the affected cell but also amplifies inflammatory responses in the surrounding tissue through the release of these potent signaling molecules [45, 46]. First observed morphologically and functionally in 1992 in *Shigella*-infected macrophages [47], this form of cell death was initially misidentified as apoptosis due to its caspase dependence, DNA fragmentation, and nuclear condensation. It remained poorly characterized until 2001, when Cookson and Brennan formally recognized it as a distinct biological process and introduced the term “pyroptosis,” marking a significant milestone in understanding its molecular mechanisms [48].

Pyroptosis pathways are primarily categorized according to their initiating caspases:

- **Canonical Pathway:** Mediated by caspase-1, which is activated within cytoplasmic inflammasome complexes in response to a variety of exogenous and endogenous stimuli.
- **Non-canonical Pathway:** Mediated by caspase-4 and -5 in humans or caspase-11 in mice, which directly detect cytosolic threats such as bacterial LPS [49, 50].
- **Other Pathways:** Emerging evidence indicates roles for caspase-3 and caspase-8 in triggering pyroptosis, often *via* cleavage of specific gasdermin (GSDM) family proteins [51].

Regardless of the initiating pathway, pyroptosis execution converges on the formation of pores in the plasma membrane by cleaved GSDM proteins [52]. Consequently, elucidating its molecular mechanisms is critically important. The following section outlines the key proteins and signaling cascades involved in the regulation of pyroptosis, as illustrated in Fig. (**1**).

### 4.1 Pyroptosis Key Proteins

#### 4.1.1 NLRP3

Identified inflammasomes include members of the NLR family and the PYHIN family, both named according to their respective scaffold-forming receptor proteins [53, 54]. Inflammasomes universally consist of a receptor, adapter, and effector. Upon activation by infection or stress, they engage in innate immune defense mechanisms *via* PAMP/DAMP recognition [55]. The classical pyroptosis pathway of inflammasome activation is typically initiated by NOD-like receptors (NLRs), with stimuli such as pathogen invasion and cellular stress triggering the initial activation. This leads to transcriptional upregulation of NLRP3, followed by activation of the NLRP3 inflammasome, culminating in the cleavage and activation of caspase-1 [56].

Structurally, NLRP3 includes three domains: the N-terminal caspase activation and recruitment domain (CARD) or Pyrin domain (PYD), the central domain (NAIP, CIITA, HET-E, and TP1), and the C-terminal leucine-rich repeat (LRR). Functionally, these domains mediate downstream protein interactions, autoinhibition, or receptor recognition [54, 57]. In the second activation step, inflammasome assembly occurs in response to changes in intracellular conditions, including increased Ca^2+^ levels and ATP release. This promotes NLRP3 oligomerization and recruitment of ASC, which contains a CARD, ultimately forming a fully assembled inflammasome [58].

#### 4.1.2 Caspases

Caspases, encoded by *CASP* genes, constitute an evolutionarily conserved family of cysteine proteases that play a central role in mediating programmed cell death—including apoptosis and pyroptosis—as well as inflammatory responses [59]. In multicellular organisms, caspase activation occurs within macromolecular signaling complexes, where aggregation of inactive proenzymes facilitates proximity-induced autocatalytic cleavage and maturation. This activation represents a fundamental mechanism of the innate immune system and serves as a critical first line of defense against pathogens [60].

To date, 14 types of caspases have been identified and can be categorized based on their involvement in different cellular physiological processes [61, 62]. Among them, caspase-1 mediates classical pyroptosis and is activated by the inflammasome, a large multiprotein complex typically composed of PRRs, caspase-1, and an adapter protein [63]. Caspase-4/5/11 mediate the non-classical pyroptosis pathway induced by LPS, leading to plasma membrane rupture, promoting K^+^ efflux, and inducing pyroptosis [64]. Caspase-3 is generally regarded as the key executor of apoptosis and can be activated by factors such as TNF-α and chemotherapeutic drugs. Caspase-8, an important initiator of apoptosis, also participates in pyroptosis, thereby linking different types of cell death [65, 66].

#### 4.1.3 GSDMs

Gasdermins (GSDMs) represent a multifunctional family of proteins belonging to the gastric mucosal defense-related protein family. These proteins are widely expressed across various cell types and tissues and serve as key mediators of pyroptosis. They were initially identified in the gastrointestinal tract and skin of mice [67, 68]. GSDMs are encoded by six homologous genes in humans. GSDMA-E share two domains: the GSDM-C terminal domain and the GSDM-N terminal domain. The release of the active GSDM-N terminal domain, which causes membrane perforation and leakage of cellular contents, occurs only when the GSDM-C terminal domain is proteolytically removed [69-71].

A key function of GSDMs is their ability to induce cell death and inflammation, particularly GSDMD, which plays a major role in inflammasome signaling and pyroptosis [72]. Caspase-1/4/5/11 can activate GSDMD, triggering the release of the active GSDMD-N-terminal fragment, while GSDME primarily mediates pyroptosis through cleavage and activation by apoptotic caspase-3, thereby releasing the GSDME-N-terminal fragment. GSDMC mediates pyroptosis through activation by caspase-8 [73, 74].

### 4.2. Pyroptosis Pathway

#### 4.2.1 Canonical Pyroptosis Pathways

The classical pathway involves NLRP3 inflammasome-mediated activation of caspase-1, which subsequently cleaves GSDMD, leading to cell membrane perforation and the release of inflammatory factors [75]. This process is initiated when DAMPs or PAMPs stimulate cells. These signals are recognized by PRRs, activating NF-κB and triggering inflammasome assembly [76].

In the next stage, inflammation is activated. The NLRP3 inflammasome is the most well-characterized classical pathway and consists of the receptor NLRP3, the linker protein ASC, and the effector protein caspase-1. NLRP3 binds to the N-terminal PYD domain of ASC through its own PYD domain. ASC then recruits procaspase-1 through its C-terminal CARD domain to form a complex. Procaspase-1 undergoes autocleavage within the activated inflammasome, generating active caspase-1 [77, 78].

Caspase-1 then mediates the maturation of pro-inflammatory cytokines by cleaving pro-IL-1β and pro-IL-18 into their active forms for extracellular release. Caspase-1 also cleaves GSDMD, liberating its N-terminal fragment (GSDMD-N). GSDMD-N binds to membrane phospholipids, forming non-selective pores (10-15 nm in diameter). In the terminal stage of pyroptosis, these pores cause ion imbalance, cellular swelling, membrane rupture, and the release of intracellular contents (including IL-1β and IL-18), thereby triggering a robust inflammatory response [79-81].

#### 4.2.2 Non-canonical Pyroptosis Pathways

Unlike the canonical pathway, the non-canonical pyroptosis pathway relies on the activation of caspase-4/5 (in humans) or caspase-11 (in mice). These caspases directly recognize LPS from Gram-negative bacteria; LPS binds to the CARD domain of caspase-4/5/11 *via* its lipid A moiety, inducing oligomerization and auto-activation. Activated caspase-4/5/11 then cleaves GSDMD, releasing the GSDMD-N fragment to form membrane pores [82, 83].

In this pathway, pro-IL-1β and pro-IL-18 cannot be processed directly. Additional mechanisms are required:

**First**, indirect activation of the NLRP3 inflammasome. Activated caspase-4/5/11 induces the opening of the Pannexin-1 channel, promoting ATP release and subsequent activation of P2X7 receptors. This cascade results in caspase-1 activation and initiation of the caspase-1-dependent pyroptosis pathway [84-86].

**Second**, amplification of caspase-1 activity. Activation of the non-canonical pathway enhances caspase-1 maturation, thereby strengthening inflammatory signaling in NASH [87].

#### 4.2.3 Other Pyroptosis Pathways

Pyroptosis can also be triggered by other Gasdermin (GSDM) family members (including GSDME, GSDMB, and GSDMC) and atypical proteases (such as caspase-3/8). Caspase-3 was previously known primarily as an executor of apoptosis [51, 88]. Activated caspase-3 cleaves GSDME, releasing its N-terminal fragment to induce pyroptosis [89]. The expression level of GSDME determines the dominant mode of cell death: high expression favors pyroptosis, whereas low expression favors apoptosis [90].

Similarly, caspase-8 is a key promoter of apoptosis and can cleave GSDMC. Although the full biological function of GSDMC remains unclear, caspase-8-mediated cleavage generates the N-terminal domain of GSDMC, forming membrane pores and inducing pyroptosis. This occurs under hypoxic conditions or following TNF-α stimulation, where TNF-α activates caspase-8 to cleave GSDMC [91-93].

Additionally, cytotoxic lymphocytes secrete granzyme A (GZMA), which directly cleaves GSDMB to induce pyroptosis in target cells, while granzyme B (GZMB) cleaves GSDME and cooperates with caspase-3 to amplify pyroptotic signaling [94, 95]. GSDM proteins may also be directly activated by bacterial toxins [96].

## 5 THE NETWORK CONNECTION OF NRF2 WITH NAFLD AND PYROPTOSIS

### 5.1 Introduction to NRF2

NRF2 is a crucial transcription factor that plays a major role in cellular defense, particularly against oxidative and xenobiotic stress [89]. NRF2 function is tightly regulated by Keap1; the Kelch domain of Keap1 binds to the high-affinity (ETGE) and low-affinity (DLG) motifs within the Neh2 domain of NRF2 [90, 91]. Under basal conditions, Keap1 sequesters NRF2 in the cytoplasm within a stable complex, maintaining low NRF2 levels and preventing its activation.

Upon exposure to oxidative stress or other stimuli, NRF2 dissociates from Keap1, translocates into the nucleus, and binds to antioxidant response elements (AREs). These DNA sequences in the promoter regions of target genes initiate the expression of antioxidant and detoxification enzymes [92, 93].

As a key regulator of anti-inflammatory and antioxidant defenses, NRF2 enhances cellular antioxidant capacity and protects against oxidative injury [94]. Given that oxidative stress is a major driver of acute and chronic liver injury, NRF2 activation can alleviate both oxidative stress and hepatic damage [95]. Although close and complex interactions exist between the mechanisms by which NRF2 inhibits pyroptosis, further investigation is warranted.

### 5.2 The Network Connection Between NRF2 and NAFLD

NRF2 is a central regulator of cellular redox homeostasis and plays a protective role in NAFLD and its progression to NASH, liver fibrosis, and hepatocellular carcinoma [97, 98]. Studies have reported increased hepatic NRF2 expression after 8 and 12 weeks of HFD feeding, with significantly higher NRF2 levels in both NAFL and NASH groups compared to controls. NRF2 overexpression was greater in NASH than in NAFL. Pearson analysis demonstrated a strong correlation between NRF2 expression and hepatic steatosis and inflammation [99].

Selective disruption of the NRF2-Keap1 interaction resulted in NASH resolution and reduced liver fibrosis in mice [100]. NRF2 acts as a transcription factor regulating cellular redox status [101]. Following the “first hit,” hepatocyte-specific NRF2 deficiency exacerbates insulin resistance and promotes hepatic lipid accumulation [22, 102]. Because IR is the primary driver of NAFLD initiation and is closely associated with disordered lipid metabolism, targeting IR and restoring hepatic lipid homeostasis is essential for preventing NAFLD onset [103].

Studies show that compared with NRF2-LoxP control mice, liver-specific NRF2 knockout mice develop less steatosis, IR, and TG accumulation after HFD feeding, changes typically observed during the early stage of NAFLD [104].

Various natural herbal extracts (such as honokiol, curcumin, and apigenin) significantly improve IR and reduce TG accumulation by activating AMPK or PPARγ pathways and modulating the Keap1-NRF2-ARE axis [105-107]. NRF2 regulates lipid metabolism genes; NRF2 deficiency activates SREBP-1c, promotes fatty acid synthesis (*e.g.*, FASN, ACCα), and contributes to hepatic lipid accumulation. It also maintains autophagy *via* genes such as LAMP1, enhances lipolysis, and suppresses ferroptosis through upregulation of SLC7A11 and GPX4.

NRF2 reduces oxidative stress and inflammation by inhibiting NF-κB and TGF-β1/Smads pathways. MicroRNAs such as miR-144 and miR-200a target Keap1 to modulate NRF2, offering potential therapeutic approaches for NAFLD [108, 109]. However, deeper mechanistic understanding of NRF2-mediated NAFLD improvement is still lacking.

IR accompanied by hyperglycemia and increased de novo lipogenesis constitutes the “first hit,” which triggers the “second hit,” including oxidative stress, inflammation, and fibrosis [110]. ROS accumulation activates the NLRP3 inflammasome, leading to caspase-1 activation, GSDMD cleavage, and pyroptosis. During cancer treatment, certain drugs can induce NRF2 dissociation from Keap1, enabling NRF2 nuclear translocation and ARE binding [111, 112]. Activation of the NRF2/ARE pathway upregulates antioxidant enzymes and detoxification factors, reducing oxidative stress. Because excessive oxidative stress activates pyroptosis, the NRF2/ARE pathway may indirectly inhibit pyroptosis through its antioxidative effects [113, 114].

In the study by Liu *et al.*, mice fed an MCD diet to induce NASH were treated with or without scoparone for 4 weeks. Scoparone affected autophagy in macrophages, reduced LPS-induced autophagosome accumulation, lowered ROS production, and attenuated inflammatory responses in RAW264.7 cells [115]. *Zanthoxylum bungeanum* (AZB) also improved NAFLD by regulating gut microbiota and activating AMPK/NRF2 signaling, thereby reducing body weight, reversing hepatic lesions and fat accumulation, and improving oxidative stress in HFD-fed mice [116].

In summary, NRF2 is closely associated with NAFLD initiation and progression, and further in- depth research holds significant scientific value.

### 5.3 The Network Connection Between NRF2 and Pyroptosis

NRF2 is a key transcription factor that protects cells from oxidative stress. Upon exposure to ROS or electrophilic stimuli, NRF2 dissociates from Keap1, enters the nucleus, binds to AREs, and activates antioxidant and detoxification genes [117].

NRF2 attenuates pyroptosis primarily by suppressing oxidative stress. After activation, NRF2 promotes the expression of antioxidant genes such as HO-1, NQO1, and SOD (Fig. **2**). Because ROS is a major trigger of NLRP3 inflammasome activation, NRF2 reduces ROS levels, thereby inhibiting NLRP3 inflammasome assembly, caspase-1 activation, and GSDMD cleavage [118, 119].

NRF2 suppresses pro-inflammatory pathways such as NF-κB and downregulates pyroptosis-related molecules including NLRP3, pro-IL-1β, and pro-IL-18. Its target gene HO-1 generates metabolites that directly inhibit NLRP3 inflammasome activity and reduce caspase-1-dependent pyroptosis. The NRF2/HO-1 pathway forms an essential antioxidant defense system; HO-1 degrades heme to produce bilirubin and carbon monoxide, both of which exert antioxidant effects. Bilirubin scavenges ROS such as superoxide anion and hydrogen peroxide [120, 121].

Activation of the NRF2/HO-1 pathway inhibits key inflammatory factors and reduces chemokine-mediated leukocyte aggregation. Consequently, it suppresses NLRP3 expression and attenuates inflammation, thereby regulating pyroptosis [122-124].

In one study, 61 rats were divided into six groups (two controls, cisplatin nephrotoxicity, curcumin, liraglutide, and co-treatment groups). Cisplatin induced acute kidney injury characterized by inflammation, oxidative imbalance, apoptosis, and pyroptosis. The NRF2/HO-1 axis and GSK-3β played major roles in nephroprotection [125].

NRF2 also indirectly regulates pyroptosis execution by inhibiting upstream inflammasomes. It blocks caspase-1/4/11-mediated GSDMD cleavage, reducing membrane pore formation and IL-1β/IL-18 release. Some studies suggest NRF2 may regulate specific microRNAs targeting GSDMD mRNA, potentially affecting GSDMD protein levels, although evidence remains limited [126].

Accumulating evidence suggests NRF2 modulates signaling molecules by inhibiting NF-κB nuclear translocation, decreasing transcription of NLRP3 and pro-inflammatory cytokines. NRF2 activation may also influence NLRP3 activity through effects on phosphorylation and other post-translational modifications [127-129].

In a hypoxia-reoxygenation model of renal tubular epithelial cells, SalB pretreatment increased nuclear NRF2 accumulation and significantly inhibited oxidative stress, inflammatory cytokines, NLRP3 activation, and pyroptosis [130]. Wang *et al.* established an AAA mouse model and demonstrated that cryptotanshinone (CTS) upregulated NRF2 target genes, particularly *Hmox-1*, thereby preventing VSMC pyroptosis mediated by NLRP3 and GSDMD. CTS showed promising therapeutic effects by modulating the Keap1-NRF2-GSDMD axis in VSMCs [131].

A deeper understanding of these interconnected signaling pathways is essential for clarifying disease mechanisms and identifying novel therapeutic targets.

## 6 THE DRUG INHIBITS PYROPTOSIS BY ACTIVATING NRF2 AND TREATS NAFLD

As summarized above, activation of NRF2 induces the expression of antioxidant genes, thereby mitigating oxidative stress, an important contributor to liver damage in NAFLD. Through the upregulation of antioxidant enzymes and detoxification factors *via* the NRF2/ARE pathway, cellular oxidative stress levels are effectively reduced. This reduction in oxidative stress contributes to the suppression of pyroptosis. Additionally, inflammation plays a significant role in the pathogenesis of NAFLD. NRF2 suppresses the expression of the inflammasome component NLRP3, thereby attenuating hepatic inflammation and pyroptosis. Consequently, pharmacological activation of NRF2-related pathways may serve as a potential therapeutic strategy for NAFLD through the modulation of pyroptosis. Table **1** summarizes the relevant drugs and their mechanisms of action.

The food-derived compound apiin (API) alleviates obesity-induced metabolic syndrome by acting as a PPARγ modulator. It reduces HFD-induced liver injury and hepatic lipid accumulation in mice. API suppresses NLRP3 inflammasome activation and the release of IL-1β and IL-18. It also inhibits xanthine oxidase activity, lowering uric acid and ROS production. Additionally, API influences NRF2, a key regulator of lipid metabolism that controls antioxidant gene expression through ARE [107, 132].

Sulforaphane (SFN), an isothiocyanate derived from broccoli sprout extract, exhibits antioxidant, anti-inflammatory, and chemopreventive effects. Oral administration of SFN reduces liver steatosis and lipid levels in high-fat diet-fed mice. These effects are linked to the inhibition of NLRP3 inflammasome activation by SFN. The antioxidant activity of SFN is associated with the activation of the NRF2-ARE pathway. SFN also decreases the expression of inflammatory markers such as ASC, caspase-1, and IL-1β [119, 133].

Melatonin (MT) is a neurohormone secreted by the pineal gland that regulates sleep, circadian rhythms, and neuroendocrine functions. In an experimental model, mice were treated with TMT (2.8 mg/kg) and MT (10 mg/kg) for 7 days. TMT induces oxidative stress by regulating NLRP3 and ASC genes, disrupting the Keap1-NRF2 pathway, and reducing antioxidant enzymes. MT treatment alleviates these adverse effects caused by TMT [134-136].

4-Acetylquinolinol B (4-AAQB), a natural coquinone derivative extracted from cinnamon, inhibits inflammatory responses, ER stress, and NLRP3 inflammasome activation in both *in vitro* and *in vivo* models. While 4-AAQB attenuated LPS-induced proinflammatory cytokines and potentiated DC immunity while elevating NRF2/SIRT1 levels in living tissues, it specifically enhanced NRF2 accumulation and HO-1 levels in LPS-treated RAW264.7 cells *in vitro* [137].

Baicalin (BA), a flavonoid glycoside from *Radix baicalensis*, is used to treat NASH. It improves FFA-induced damage and cell death in HepG2 hepatocytes. In NASH mice, BA reduces liver lipid accumulation and inflammation. This effect is linked to increased NRF2/HO-1 expression and reduced levels of pyroptosis-related proteins such as NLRP3, GSDMD, and IL-1β. Further analysis confirms BA’s inhibitory effect on liver pyroptosis [138, 139].

Magnolol (MG), the main active compound of *Magnolia officinalis*, has diverse biological activities. It was tested in tyloxapol-induced hyperlipidemic rats and palmitic acid-stimulated HepG2 cells. MG reduced the expression of IL-1β, NLRP3, ASC, and caspase-1 and promoted NRF2 nuclear translocation and HO-1 expression in treated rats. It inhibits NLRP3 inflammasome activation by restoring autophagy, leading to enhanced HO-1 signaling, improved oxidative stress, and reduced inflammation [140].

Auranofin, a drug for rheumatoid arthritis, shows potential in treating metabolic syndrome and NAFLD through immune regulation. In C57BL/6 mice, it reduced HFD-induced hepatic steatosis. It also suppressed inflammasome-related markers including NLRP3, caspase-1, IL-1β, and IL-18. Additionally, auranofin lowered NOX4 expression, a major source of oxidative stress, and reduced PPARγ levels, a key regulator of adipogenesis [141].

Benzyl isothiocyanate (BITC), found in cruciferous vegetables, has antioxidant, anti-inflammatory, anticancer, and anti-obesity effects. In an HFCCD-induced steatohepatitis mouse model, BITC suppressed NLRP3 inflammasome activation in LPS-stimulated Kupffer cells. This effect was mediated by enhanced PKA-dependent NLRP3 ubiquitination, leading to reduced IL-1β production. As a result, BITC attenuated IL-1β-induced hepatic inflammation in hepatocytes. It also improved systemic IR [142, 143].

Berberine, an isoquinoline alkaloid from medicinal plants, shows therapeutic potential for NAFLD. It reduced body weight, liver weight, and hepatic steatosis in high-fat diet-induced NAFLD rats. Berberine improved lipid accumulation and lowered ROS, lipid peroxides, and TNF-α levels while suppressing NF-κB p65 phosphorylation. It also inhibited NLRP3 inflammasome activation and reduced GSDMD-NT expression. These effects collectively suppress hepatocyte pyroptosis, mediated by NRF2 activation [144, 145].

Curcumin, a polyphenolic compound from *Curcuma longa*, has broad pharmacological effects and is widely studied. It exerts hepatoprotective effects by inhibiting NLRP3 inflammasome components (NLRP3, caspase-1, GSDMD) at both mRNA and protein levels. It suppresses proinflammatory cytokines, lipid peroxidation, and PI3K/Akt signaling activation. Curcumin also inhibits hepatic stellate cell activation and enhances antioxidant responses *via* NRF2 signaling and SOD expression. These effects are attributed to its ability to scavenge reactive oxygen species through phenolic and β-diketone structures [106, 146, 147].

Danshen Zexie Decoction (DZD), a modified Chinese herbal formula mainly derived from *Salvia miltiorrhiza*, exhibits lipid-regulating effects. In a high-fat diet-induced NAFLD rat model, DZD improved lipid metabolism disorders and liver injury by reducing lipid accumulation, oxidative stress, and inflammation. It inhibited hepatocyte pyroptosis, as shown by reduced serum IL-1β and IL-18 levels and suppressed NLRP3 inflammasome activation (lower NLRP3, caspase-1, GSDMD, and GSDMD-NT expression). DZD also activated NRF2-HO-1 signaling, which contributed to the suppression of the ROS/NLRP3/IL-1β pyroptotic pathway [148, 149].

NRF2 regulates autophagy-related genes and promotes autophagy, which helps degrade metabolic waste and toxic substances, reducing liver burden. Impaired autophagy in NAFLD can lead to lipid accumulation and cell damage. NRF2 enhances autophagy to reduce the occurrence of pyroptosis. Here are the relevant drugs for treating NAFLD by promoting autophagy.

Cannabidiol (CBD), a major non-psychotropic compound from *Cannabis sativa*, reduces hepatic inflammation in HFD-fed mice. It works through partial inhibition of NF-κB signaling and enhanced suppression of NF-κB activation. CBD also inhibits the NF-κB/NLRP3 axis, reduces oxidative stress, and activates hepatoprotective autophagy. These mechanisms provide significant protection against fatty liver disease development [83].

Exenatide (Exe), the first GLP-1 receptor agonist, improves glycemic control and lipid metabolism. In NAFLD mice, Exe reduced body and liver weight and lipid accumulation while increasing autophagosome formation. It also suppressed NLRP3 and IL-1β expression. Exe exerts hepatoprotective effects by enhancing autophagy and mitophagy, thereby reducing oxidative stress and NLRP3 inflammasome activation [150].

Saponins, key bioactive compounds from *Panax notoginseng*, have broad pharmacological effects. In a 16-week diet-induced NAFLD mouse model, saponin treatment initiated after 9 weeks improved hepatic steatosis, fibrosis, and inflammation. These effects were linked to anti-inflammatory activity and autophagy modulation in Kupffer cells. Saponins show therapeutic potential for NAFLD through multiple mechanisms [151].

Pterostilbene (PTE) alleviates NAFLD by activating NRF2 to enhance autophagy and reduce oxidative stress. It significantly reduced lipid accumulation in HepG2 cells and mouse models. PTE promoted NRF2 nuclear translocation, increased PPAR-α and HO-1 expression, and activated AMPK signaling. It also suppressed mTORC1 and SREBP-1c while upregulating PI3K and autophagy-related genes (ATGs), as shown by an increased LC3-II/LC3-I ratio. NRF2 knockdown abolished all therapeutic effects, confirming NRF2’s essential role in PTE’s mechanism [152].

## CONCLUSION

NAFLD, one of the world’s most prevalent chronic hepatic disorders, involves complex pathogenesis driven by oxidative stress, inflammation, lipid metabolic dysregulation, and programmed cell death. Recent research highlights the NRF2 signaling pathway as a central regulator of oxidative and inflammatory responses, while pyroptosis, an inflammatory form of programmed cell death, has emerged as critically implicated in NAFLD progression. Current evidence indicates that therapeutic strategies targeting pyroptosis inhibition through NRF2 pathway activation hold significant promise. However, mechanistic elucidation and clinical translation still face substantial challenges. Although NRF2 is protective in the early stages of NAFLD (simple steatosis), it may exacerbate fibrosis by promoting hepatic stellate cell survival in advanced fibrotic stages. Current studies lack staged dynamic analyses; for example, most animal models simulate only a 12-week high-fat diet, which does not encompass the full progression from NASH to hepatic fibrosis.

Existing studies have preliminarily demonstrated that NRF2 activation can inhibit pyroptosis by limiting ROS accumulation, regulating inflammasome activity (such as NLRP3), and reducing GSDMD cleavage. However, the interaction network between NRF2 and pyroptotic signaling pathways requires further refinement. It remains unclear whether NRF2 directly regulates GSDMD/GSDME protein activity. Similarly, whether NRF2 indirectly affects pyroptosis through the regulation of ferroptosis has yet to be verified. In NAFLD, lipid-lowering hepatoprotective agents inhibit ferroptosis through activation of the NRF2-SLC7A11-GPX4 axis, but the interaction between this pathway and pyroptosis (*e.g.*, whether lipid peroxidation triggers NLRP3 activation) has not been clarified. The priority and synergistic mechanisms of these two forms of programmed cell death during NAFLD progression remain unaddressed.

Moreover, the specific regulation of NRF2 downstream targets and how NRF2-induced antioxidant enzymes interfere with the expression of pyroptosis-related proteins (such as caspase-1 and IL-1β) are still unclear. Single-cell sequencing or spatial transcriptomics will be necessary to analyze pathway differences across distinct liver cell subsets. In addition, the synergistic interactions between NRF2 and other metabolic regulators, such as AMPK and PPARγ, may provide new targets for pyroptosis inhibition. In the tumor microenvironment, high NRF2 expression supports cancer cell survival by maintaining GSH pools that inhibit cell death. This raises concerns that NAFLD treatments targeting NRF2 may inadvertently increase hepatocellular carcinoma risk; however, long-term safety data are lacking. Furthermore, although NRF2 activation blocks KEAP1-mediated degradation, systemic administration can result in off-target effects. Liver-targeted delivery systems (*e.g.*, exosomes or nanocarriers) remain in the experimental stage.

At present, very few clinical treatments for NAFLD specifically target the NRF2 pathway to inhibit pyroptosis. Although DZD, Exenatide, and Berberine can be used for NAFLD management, Baicalin is used as an adjuvant therapy for chronic active hepatitis, the clinical application of Melatonin has expanded from sleep regulation to fields such as metabolism, neurology, oncology, and inflammation, Auranofin is in phase III clinical trials for rheumatoid arthritis, Curcumin is used for sepsis treatment, and Cannabidiol is effective for epilepsy. However, most other compounds remain in preclinical testing, and therapeutic strategies for NAFLD are still under active investigation. As a central hub connecting oxidative stress, inflammation, and cell death, the NRF2 pathway offers a promising intervention strategy for NAFLD. Nevertheless, translating basic research into clinical practice will require deep integration across multidisciplinary collaboration, technological innovation, and translational medicine. In the next decade, with advancements in precision medicine and targeted delivery technologies, NRF2 activators are expected to become an important focus in NAFLD drug development, potentially providing safer and more effective treatments for hundreds of millions of patients worldwide.

## Figures and Tables

**Fig. (1):**
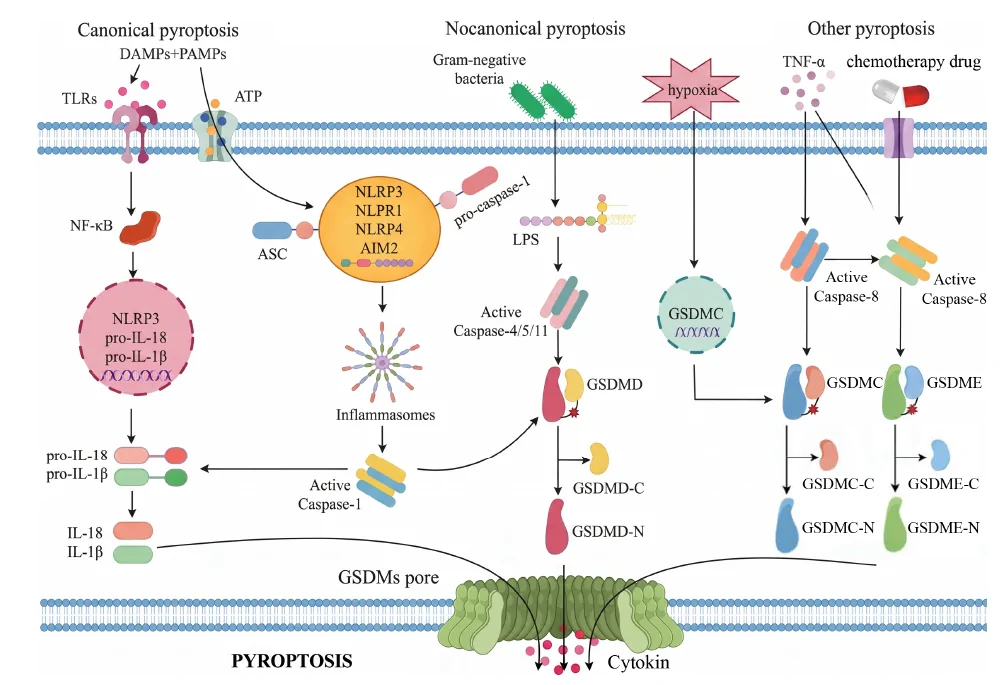
Overview of pyroptosis signaling pathways. Classical pyroptosis pathway initiation involves DAMPs or PAMPs activating PRRs, which trigger NF-κB signaling and inflammasome assembly. The NLRP3 inflammasome consists of NLRP3, ASC, and caspase-1, and activates procaspase-1 through domain interactions. Activated caspase-1 processes pro-IL-1β and pro-IL-18 into mature cytokines and cleaves GSDMD to produce GSDMD-N. Non-classical pyroptosis pathway activation relies on caspase-4/5 (in humans) or caspase-11 (in mice), which directly bind to the lipid A component of Gram-negative bacterial LPS, inducing oligomerization and autoactivation. Activated caspase-4/5/11 then cleaves GSDMD, releasing GSDMD-N to form membrane pores. Unlike the classical pathway, IL-1β and IL-18 are not directly processed in this pathway. Other Gasdermin family members (*e.g.*, GSDME, GSDMB, GSDMC) and atypical proteases like caspase-3/8 can also induce pyroptosis. Activated caspase-3 cleaves GSDME, releasing its N-terminal fragment to trigger pyroptosis, with cell fate determined by GSDME expression levels. Caspase-8 cleaves GSDMC under hypoxic conditions or TNF-α stimulation, leading to pyroptosis. **Abbreviations:** DAMPs:damage-associated molecular patterns. PAMPs: pathogen-associated molecular patterns. TLR.Toll-Like Receptor. ATP: adenosine triphosphate.NF-kB: nuclear factor kappa B. LPS: lipopolysaccharide. GSDMB/C/D/E: gasdermin B/C/D/E. IL: interleukin. LPS: lipopolysaccharides. NLRP3: NLR family pyrin domain-containing 3. ASC: apoptosis-associated speck-like protein containing a CARD.

**Fig. (2):**
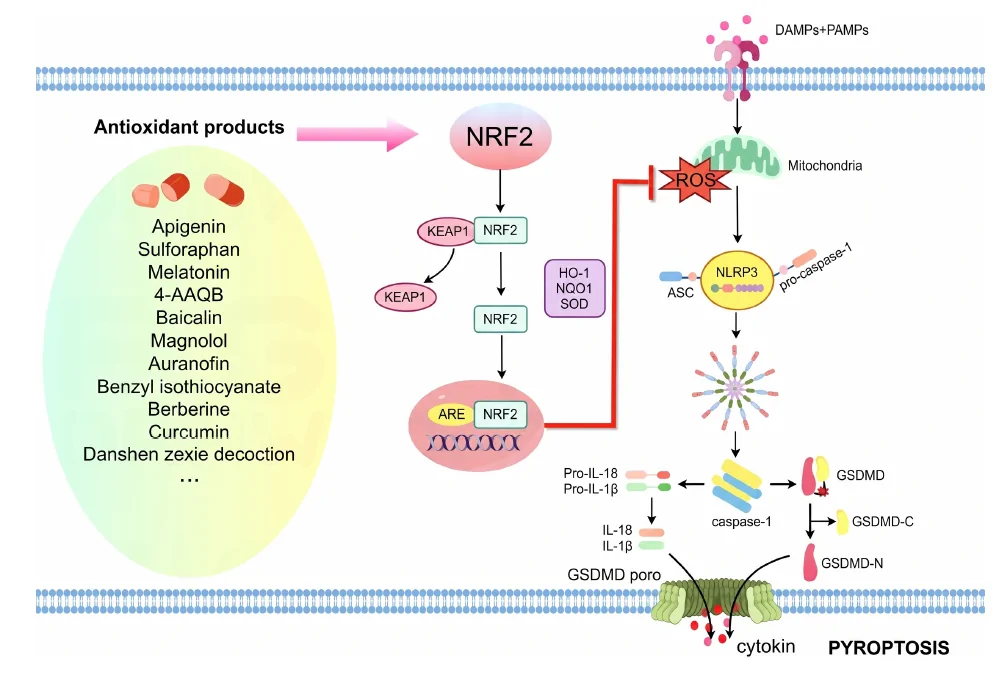
Antioxidant products improve pyroptosis by inhibiting the NRF2 pathway. NRF2 dissociates from KEAP1 upon exposure to ROS or electrophilic stimuli. Antioxidant products enhance NRF2 activity; these compounds are essential for inflammasome activation and pyroptosis induction, which reduces ROS levels. Thus, antioxidant agents may suppress inflammation *via* NRF2 activation.

**Table 1:** NRF2-based therapeutic strategy to inhibit pyroptosis in NAFLD.

**Inhibitors/Compound**	**Source**	**Type of Molecule**	**Mechanism**	**Vivo Experiment**	**Preclinical**	**Clinical**	**References**
Apigenin (API)	Plants	naturally occurring bioflavonoid	Inhibiting NLRP3 inflammasome activation and release of inflammatory cytokines IL-1β and IL-18	Hepatic cell	Mice	\	[107, 132]
Sulforaphane (SFN)	Plants broccoli sprouts	Extract	Inhibiting NLRP3 inflammasome activation	\	Mice	\	[119, 133]
Melatonin	pineal gland	hormone	Inhibiting the expressions of NLRP3, ASC, Cas1, and IL-1β	\	Mice		[134-136]
4-acetylantroquinonolB(4-AAQB)	Plants Antrodia cinnamomea	mycelia	Inhibiting NLRP3 inflammasome activation	RAW264.7	Mice	\	[137]
Baicalin	Plants Scutellaria baicalensi	flavone glycoside	Inhibiting the expression of NLRP3, GSDMD, and IL-1β expression	HepG2 cell	Mice	viral hepatitis	[138, 139, 153]
Magnolol	Plants Magnolia officinalis	bioactive compound derived	Inhibiting NLRP3 inflammasome activation	HepG2 cell	Rat	\	[140]
Auranofin	synthetic	protease inhibitor cocktail,	Inhibiting the expressions of IL-1β,IL-18, caspase-1, and NLRP3	\	Mice	RA(rheumatoid arthritis)	[141, 154]
Benzyl isothiocyanate (BITC)	Plants cruciferous vegetables	organosulfur phytochemical derived	isothiocyanate Inhibiting the NLRP3 inflammasome activation by enhancing the PKA-dependent ubiquitination of NLRP3	Kupffer cells	Mice	\	[142, 143]
Berberine	Plants	isoquinoline alkaloid originating	Inhibiting NLRP3 and GSDMD-N expression as well as caspase-1 activity	AML12 cell	Mice	NAFLD/NASH; Type 2 diabetes	[144, 145, 155]
Curcumin	Plants	Polyphenol	Inhibiting NLRP3 expression as well as caspase-1 activity	\	Rat; Mice	pyohemia	[106, 146, 147, 156]
Danshen zexie decoction	Plants	Chinese herbal medicine	Suppressed the expressions of NLRP3, caspase-1, GSDMD, and GSDMD-N	\	Mice	NAFLD	[148, 157]
Cannabidiol	Plants marijuana	an abundant non-psychoactive constituent	inhibited the NF-κB p65 nuclear translocation and the activation of the NLRP3 inflammasome	RAW264.7 Kupffer cells	Mice	epilepsy	[83, 158]
Exenatide	polypeptide	glucagon-like peptide-1 (GLP-1) receptor agonist (GLP-1RA)	Inhibiting the NLRP3 inflammasome in Mice	\	Mice	NAFLD/NASH; Type 2 diabetes	[150, 159, 160]
Ginseng Saponin	Plants	Saponin	Inhibiting NLRP3 inflammasome activation	Kupffer cells	Mice	\	[151]
Pterostilbene	Plants	an active compound	-	HepG2 cells	Mice	asthma	[152, 161]

## LIST OF ABBREVIATIONS

NAFLD: Non-alcoholic Fatty Liver Disease
NASH: Non-alcoholic Steatohepatitis
NRF2: Nuclear Factor Erythroid 2-related Factor 2
Keap1: Kelch-like ECH-associated Protein 1
AREs: Antioxidant Response Elements
NF-κB: Nuclear Factor Kappa B
TLRs: Toll-like Receptors
CLRs: C-type Lectin Receptors

## References

[1] Lonardo A., Byrne C.D., Caldwell S.H., Cortez-Pinto H., Targher G. (2016). Global epidemiology of nonalcoholic fatty liver disease: Meta-analytic assessment of prevalence, incidence, and outcomes.. Hepatology.

[2] Pafili K., Roden M. (2021). Nonalcoholic fatty liver disease (NAFLD) from pathogenesis to treatment concepts in humans.. Mol. Metab..

[3] Ying Y., Zhang H., Yu D., Zhang W., Zhou D., Liu S. (2021). Gegen qinlian decoction ameliorates nonalcoholic fatty liver disease in rats *via* oxidative stress, inflammation, and the NLRP3 signal axis.. Evid. Based Complement. Alternat. Med..

[4] Xu H., Wan S., An Y., Wu Q., Xing Y., Deng C., Zhang P., Long Y., Xu B., Jiang Z. (2024). Targeting cell death in NAFLD: mechanisms and targeted therapies.. Cell Death Discov..

[5] Wree A., McGeough M.D., Inzaugarat M.E., Eguchi A., Schuster S., Johnson C.D., Peña C.A., Geisler L.J., Papouchado B.G., Hoffman H.M., Feldstein A.E. (2018). NLRP3 inflammasome driven liver injury and fibrosis: Roles of IL-17 and TNF in mice.. Hepatology.

[6] Aziz M., Jacob A., Yang W.L., Matsuda A., Wang P. (2012). Current trends in inflammatory and immunomodulatory mediators in sepsis.. J. Leukoc. Biol..

[7] Gao B., Tsukamoto H. (2016). Inflammation in alcoholic and nonalcoholic fatty liver disease: Friend or foe?. Gastroenterology.

[8] Wiering L., Tacke F. (2023). Treating inflammation to combat non-alcoholic fatty liver disease.. J. Endocrinol..

[9] Lee Y.A., Friedman S.L. (2022). Inflammatory and fibrotic mechanisms in NAFLD—Implications for new treatment strategies.. J. Intern. Med..

[10] Alisi A., Carpino G., Oliveira F.L., Panera N., Nobili V., Gaudio E. (2017). The role of tissue macrophage-mediated inflammation on NAFLD pathogenesis and its clinical implications.. Mediators Inflamm..

[11] Zhang D., Mao F., Wang S., Wu H., Wang S., Liao Y. (2023). Role of transcription factor Nrf2 in pyroptosis in spinal cord injury by regulating GSDMD.. Neurochem. Res..

[12] Zenkov N.K., Menshchikova E.B., Tkachev V.O. (2013). Keap1/Nrf2/ARE redox-sensitive signaling system as a pharmacological target.. Biochemistry (Mosc).

[13] Tu W., Wang H., Li S., Liu Q., Sha H. (2019). The anti-inflammatory and anti-oxidant mechanisms of the Keap1/Nrf2/ARE signaling pathway in chronic diseases.. Aging Dis..

[14] Zhou Y., Jia Z., Wang J., Huang S., Yang S., Xiao S., Xia D., Zhou Y. (2023). Curcumin reverses erastin-induced chondrocyte ferroptosis by upregulating Nrf2.. Heliyon.

[15] Cui C., Gao S., Shi J., Wang K. (2025). Gut-liver axis: The role of intestinal microbiota and their metabolites in the progression of metabolic dysfunction-associated steatotic liver disease.. Gut Liver.

[16] Li N., Wang B., Wu Y., Luo X., Chen Z., Sang C., Xiong T. (2019). Modification effects of SanWei GanJiang powder on liver and intestinal damage through reversing bile acid homeostasis.. Biomed. Pharmacother..

[17] Santos-Laso A., Gutiérrez-Larrañaga M., Alonso-Peña M., Medina J.M., Iruzubieta P., Arias-Loste M.T., López-Hoyos M., Crespo J. (2021). Pathophysiological mechanisms in non-alcoholic fatty liver disease: From drivers to targets.. Biomedicines.

[18] Han S.K., Baik S.K., Kim M.Y. (2023). Non-alcoholic fatty liver disease: Definition and subtypes.. Clin. Mol. Hepatol..

[19] Loomba R., Wong V.W.S. (2024). Implications of the new nomenclature of steatotic liver disease and definition of metabolic dysfunction-associated steatotic liver disease.. Aliment Pharmacol. Ther..

[20] Buzzetti E., Pinzani M., Tsochatzis E.A. (2016). The multiple-hit pathogenesis of non-alcoholic fatty liver disease (NAFLD).. Metabolism.

[21] Wang Z., Li S., Wang R., Guo L., Xu D., Zhang T., Xu Y., Wang W., Wang M., Gan Z., Wang X. (2020). The protective effects of the β3 adrenergic receptor agonist BRL37344 against liver steatosis and inflammation in a rat model of high-fat diet-induced nonalcoholic fatty liver disease (NAFLD).. Mol. Med..

[22] Wang Y.L., Wu J., Li R.X., Sun Y.T., Ma Y.J., Zhao C.Y., Zou J., Zhang Y.Y., Sun X.D. (2021). A double-edged sword: The Kelch-like ECH-associated protein 1-nuclear factor erythroid-derived 2-related factor 2-antioxidant response element pathway targeted pharmacological modulation in nonalcoholic fatty liver disease.. Curr. Opin. Pharmacol..

[23] Galiero R., Caturano A., Vetrano E., Cesaro A., Rinaldi L., Salvatore T., Marfella R., Sardu C., Moscarella E., Gragnano F., Calabrò P., Sasso F.C. (2021). Pathophysiological mechanisms and clinical evidence of relationship between Nonalcoholic fatty liver disease (NAFLD) and cardiovascular disease.. Rev. Cardiovasc. Med..

[24] Jin X., Qiu T., Li L., Yu R., Chen X., Li C., Proud C.G., Jiang T. (2023). Pathophysiology of obesity and its associated diseases.. Acta Pharm. Sin. B..

[25] Perdomo C.M., Garcia-Fernandez N., Escalada J. (2021). Diabetic kidney disease, cardiovascular disease and non-alcoholic fatty liver disease: A new triumvirate?. J. Clin. Med..

[26] Schwabe R.F., Luedde T. (2018). Apoptosis and necroptosis in the liver: A matter of life and death.. Nat. Rev. Gastroenterol. Hepatol..

[27] Ota T (2021). Molecular mechanisms of nonalcoholic fatty liver disease (NAFLD)/nonalcoholic steatohepatitis (NASH).. Adv Exp. Med. Biol..

[28] Jacome-Sosa M.M., Parks E.J. (2014). Fatty acid sources and their fluxes as they contribute to plasma triglyceride concentrations and fatty liver in humans.. Curr. Opin. Lipidol..

[29] Nagarajan P., Mahesh Kumar M.J., Venkatesan R., Majundar S.S., Juyal R.C. (2012). Genetically modified mouse models for the study of nonalcoholic fatty liver disease.. World J. Gastroenterol..

[30] Guo H., Callaway J.B., Ting J.P.Y. (2015). Inflammasomes: Mechanism of action, role in disease, and therapeutics.. Nat. Med..

[31] Peiseler M., Schwabe R., Hampe J., Kubes P., Heikenwälder M., Tacke F. (2022). Immune mechanisms linking metabolic injury to inflammation and fibrosis in fatty liver disease – novel insights into cellular communication circuits.. J. Hepatol..

[32] Farzanegi P., Dana A., Ebrahimpoor Z., Asadi M., Azarbayjani M.A. (2019). Mechanisms of beneficial effects of exercise training on non-alcoholic fatty liver disease (NAFLD): Roles of oxidative stress and inflammation.. Eur. J. Sport Sci..

[33] Manne V, Handa P, Kowdley KV (2018). Pathophysiology of nonalcoholic fatty liver disease/nonalcoholic steatohepatitis.. Clin. Liver Dis..

[34] Cheung E.C., Vousden K.H. (2022). The role of ROS in tumour development and progression.. Nat. Rev. Cancer.

[35] Zheng D., Liu J., Piao H., Zhu Z., Wei R., Liu K. (2022). ROS-triggered endothelial cell death mechanisms: Focus on pyroptosis, parthanatos, and ferroptosis.. Front Immunol..

[36] Chen Z., Tian R., She Z., Cai J., Li H. (2020). Role of oxidative stress in the pathogenesis of nonalcoholic fatty liver disease.. Free Radic. Biol. Med..

[37] Perico L., Remuzzi G., Benigni A. (2024). Sirtuins in kidney health and disease.. Nat. Rev. Nephrol..

[38] Łanoszka K., Vlčková N. (2023). Natural sirtuin1 activators and atherosclerosis: An overview.. Curr. Atheroscler Rep..

[39] Day C.P. (2002). Pathogenesis of steatohepatitis.. Best Pract. Res. Clin. Gastroenterol..

[40] Dallio M., Sangineto M., Romeo M., Villani R., Romano A.D., Loguercio C., Serviddio G., Federico A. (2021). Immunity as cornerstone of non-alcoholic fatty liver disease: The contribution of oxidative stress in the disease progression.. Int. J. Mol. Sci..

[41] Ashraf N.U., Sheikh T.A. (2015). Endoplasmic reticulum stress and Oxidative stress in the pathogenesis of Non-alcoholic fatty liver disease.. Free Radic. Res..

[42] Xue L., He J., Gao N., Lu X., Li M., Wu X., Liu Z., Jin Y., Liu J., Xu J., Geng Y. (2017). Probiotics may delay the progression of nonalcoholic fatty liver disease by restoring the gut microbiota structure and improving intestinal endotoxemia.. Sci. Rep..

[43] Wang B., Jiang X., Cao M., Ge J., Bao Q., Tang L., Chen Y., Li L. (2016). Altered fecal microbiota correlates with liver biochemistry in nonobese patients with non-alcoholic fatty liver disease.. Sci. Rep..

[44] Vallianou N., Christodoulatos G.S., Karampela I., Tsilingiris D., Magkos F., Stratigou T., Kounatidis D., Dalamaga M. (2021). Understanding the role of the gut microbiome and microbial metabolites in non-alcoholic fatty liver disease: Current evidence and perspectives.. Biomolecules.

[45] Sasaki M., Nakanuma Y. (2017). Stress-induced cellular responses and cell death mechanisms during inflammatory cholangiopathies.. Clin. Res. Hepatol. Gastroenterol..

[46] Chen Y., Smith M.R., Thirumalai K., Zychlinsky A. (1996). A bacterial invasin induces macrophage apoptosis by binding directly to ICE.. EMBO J..

[47] Zychlinsky A., Prevost M.C., Sansonetti P.J. (1992). Shigella flexneri induces apoptosis in infected macrophages.. Nature.

[48] Cookson B.T., Brennan M.A. (2001). Pro-inflammatory programmed cell death.. Trends Microbiol..

[49] Broz P., Dixit V.M. (2016). Inflammasomes: mechanism of assembly, regulation and signalling.. Nat. Rev. Immunol..

[50] Dong T., Liao D., Liu X., Lei X. (2015). Using small molecules to dissect non-apoptotic programmed cell death: Necroptosis, ferroptosis, and pyroptosis.. ChemBioChem.

[51] Wang Y, Gao W, Shi X, Ding J, Liu W, He H, Wang K, Shao F (2017). Chemotherapy drugs induce pyroptosis through caspase-3 cleavage of a gasdermin.. Nature.

[52] Xia X., Wang X., Cheng Z., Qin W., Lei L., Jiang J., Hu J. (2019). The role of pyroptosis in cancer: Pro-cancer or pro-“host”?. Cell Death Dis..

[53] Fink S.L., Cookson B.T. (2006). Caspase-1-dependent pore formation during pyroptosis leads to osmotic lysis of infected host macrophages.. Cell Microbiol..

[54] Liston A., Masters S.L. (2017). Homeostasis-altering molecular processes as mechanisms of inflammasome activation.. Nat. Rev. Immunol..

[55] Schroder K., Tschopp J. (2010). The inflammasomes.. Cell.

[56] Liu T., Zhou Y.T., Wang L.Q., Li L.Y., Bao Q., Tian S., Chen M.X., Chen H.X., Cui J., Li C.W. (2019). NOD-like receptor family, pyrin domain containing 3 (NLRP3) contributes to inflammation, pyroptosis, and mucin production in human airway epithelium on rhinovirus infection.. J. Allergy Clin. Immunol..

[57] Cunha L.D., Zamboni D.S. (2013). Subversion of inflammasome activation and pyroptosis by pathogenic bacteria.. Front Cell Infect. Microbiol..

[58] Li R., Xue W., Wei H., Fan Q., Li X., Qiu Y., Cui D. (2023). Research progress of pyroptosis in fatty liver disease.. Int. J. Mol. Sci..

[59] Kesavardhana S., Malireddi R.K.S., Kanneganti T.D. (2020). Caspases in cell death, inflammation, and pyroptosis.. Annu. Rev. Immunol..

[60] Van Opdenbosch N., Lamkanfi M. (2019). Caspases in cell death, inflammation, and disease.. Immunity.

[61] Sahoo G., Samal D., Khandayataray P., Murthy M.K. (2023). A review on caspases: Key regulators of biological activities and apoptosis.. Mol. Neurobiol..

[62] Shi J., Gao W., Shao F. (2017). Pyroptosis: Gasdermin-mediated programmed necrotic cell death.. Trends Biochem. Sci..

[63] Tsuchiya K., Nakajima S., Hosojima S., Thi Nguyen D., Hattori T., Manh Le T., Hori O., Mahib M.R., Yamaguchi Y., Miura M., Kinoshita T., Kushiyama H., Sakurai M., Shiroishi T., Suda T. (2019). Caspase-1 initiates apoptosis in the absence of gasdermin D.. Nat. Commun..

[64] Shi J., Zhao Y., Wang K., Shi X., Wang Y., Huang H., Zhuang Y., Cai T., Wang F., Shao F. (2015). Cleavage of GSDMD by inflammatory caspases determines pyroptotic cell death.. Nature.

[65] Wang G., Li Y., Guo Z., He Q., Liu Z., Deng B., Tanshinone I. (2024). Tanshinone I stimulates pyroptosis of cisplatin-resistant gastric cancer cells by activating the NF-κB/Caspase-3(8)/GSDME signaling pathway.. DNA Cell Biol..

[66] Fritsch M., Günther S.D., Schwarzer R., Albert M.C., Schorn F., Werthenbach J.P., Schiffmann L.M., Stair N., Stocks H., Seeger J.M., Lamkanfi M., Krönke M., Pasparakis M., Kashkar H. (2019). Caspase-8 is the molecular switch for apoptosis, necroptosis and pyroptosis.. Nature.

[67] Aglietti R.A., Dueber E.C. (2017). Recent insights into the molecular mechanisms underlying pyroptosis and gasdermin family functions.. Trends Immunol..

[68] Weindel C.G., Ellzey L.M., Martinez E.L., Watson R.O., Patrick K.L. (2023). Gasdermins gone wild: New roles for GSDMs in regulating cellular homeostasis.. Trends Cell Biol..

[69] Du T., Gao J., Li P., Wang Y., Qi Q., Liu X., Li J., Wang C., Du L. (2021). Pyroptosis, metabolism, and tumor immune microenvironment.. Clin. Transl. Med..

[70] Saeki N., Kuwahara Y., Sasaki H., Satoh H., Shiroishi T. (2000). Gasdermin (Gsdm) localizing to mouse Chromosome 11 is predominantly expressed in upper gastrointestinal tract but significantly suppressed in human gastric cancer cells.. Mamm Genome.

[71] Ding J., Wang K., Liu W. (2016). Pore-forming activity and structural autoinhibition of the gasdermin family.. Nature.

[72] Sborgi L., Rühl S., Mulvihill E., Pipercevic J., Heilig R., Stahlberg H., Farady C.J., Müller D.J., Broz P., Hiller S. (2016). GSDMD membrane pore formation constitutes the mechanism of pyroptotic cell death.. EMBO J..

[73] Zhong X., Zeng H., Zhou Z., Su Y., Cheng H., Hou Y., She Y., Feng N., Wang J., Shao F., Ding J. (2023). Structural mechanisms for regulation of GSDMB pore-forming activity.. Nature.

[74] Ruan J. (2019). Structural insight of gasdermin family driving pyroptotic cell death.. Adv. Exp. Med. Biol..

[75] Burdette B.E., Esparza A.N., Zhu H., Wang S. (2021). Gasdermin D in pyroptosis.. Acta Pharm. Sin. B..

[76] Platnich J.M., Muruve D.A. (2019). NOD-like receptors and inflammasomes: A review of their canonical and non-canonical signaling pathways.. Arch. Biochem. Biophys..

[77] Lu A., Magupalli V.G., Ruan J., Yin Q., Atianand M.K., Vos M.R., Schröder G.F., Fitzgerald K.A., Wu H., Egelman E.H. (2014). Unified polymerization mechanism for the assembly of ASC-dependent inflammasomes.. Cell.

[78] Xu J., Núñez G. (2023). The NLRP3 inflammasome: Activation and regulation.. Trends Biochem. Sci..

[79] Le Y., Guo J., Liu Z., Liu J., Liu Y., Chen H., Qiu J., Wang C., Dou X., Lu D. (2024). Calenduloside E ameliorates non-alcoholic fatty liver disease *via* modulating a pyroptosis-dependent pathway.. J. Ethnopharmacol..

[80] Mao F., Wang E., Fu L., Fan W., Zhou J., Yan G., Liu T., Li Y. (2024). Identification of pyroptosis-related gene signature in nonalcoholic steatohepatitis.. Sci. Rep..

[81] Yan H., Luo B., Wu X., Guan F., Yu X., Zhao L., Ke X., Wu J., Yuan J. (2021). Cisplatin induces pyroptosis *via* activation of MEG3/NLRP3/caspase-1/GSDMD pathway in triple-negative breast cancer.. Int. J. Biol. Sci..

[82] Pilla D.M., Hagar J.A., Haldar A.K., Mason A.K., Degrandi D., Pfeffer K., Ernst R.K., Yamamoto M., Miao E.A., Coers J. (2014). Guanylate binding proteins promote caspase-11–dependent pyroptosis in response to cytoplasmic LPS.. Proc. Natl. Acad. Sci. USA.

[83] Huang X., Feng Y., Xiong G., Whyte S., Duan J., Yang Y., Wang K., Yang S., Geng Y., Ou Y., Chen D. (2019). Caspase-11, a specific sensor for intracellular lipopolysaccharide recognition, mediates the non-canonical inflammatory pathway of pyroptosis.. Cell Biosci..

[84] Du X.S., Li H.D., Yang X.J., Li J.J., Xu J.J., Chen Y., Xu Q.Q., Yang L., He C.S., Huang C., Meng X.M., Li J. (2019). Wogonin attenuates liver fibrosis *via* regulating hepatic stellate cell activation and apoptosis.. Int. Immunopharmacol..

[85] Li S.J., Liu A.B., Yu Y.Y., Ma J.H. (2024). The role and mechanism of pyroptosis and potential therapeutic targets in non-alcoholic fatty liver disease (NAFLD).. Front Cell Dev. Biol..

[86] Yuan Y.Y., Xie K.X., Wang S.L., Yuan L.W. (2018). Inflammatory caspase-related pyroptosis: mechanism, regulation and therapeutic potential for inflammatory bowel disease.. Gastroenterol. Rep..

[87] Zhu Y., Zhao H., Lu J., Lin K., Ni J., Wu G., Tang H. (2021). Caspase-11–mediated hepatocytic pyroptosis promotes the progression of nonalcoholic steatohepatitis.. Cell Mol. Gastroenterol. Hepatol..

[88] Hearps A.C., Burrows J., Connor C.E., Woods G.M., Lowenthal R.M., Ragg S.J. (2002). Mitochondrial cytochrome c release precedes transmembrane depolarisation and caspase-3 activation during ceramide-induced apoptosis of Jurkat T cells.. Apoptosis.

[89] Jiang M., Qi L., Li L., Li Y. (2020). The caspase-3/GSDME signal pathway as a switch between apoptosis and pyroptosis in cancer.. Cell Death Discov..

[90] Mao X., Li J., Xie X., Chen S., Huang Q., Mu P., Jiang J., Deng Y. (2022). Deoxynivalenol induces caspase-3/GSDME-dependent pyroptosis and inflammation in mouse liver and HepaRG cells.. Arch. Toxicol..

[91] Pang J., Vince J.E. (2023). The role of caspase-8 in inflammatory signalling and pyroptotic cell death.. Semin. Immunol..

[92] Deng J., Qin L., Qin S., Wu R., Huang G., Fang Y., Huang L., Zhou Z. (2024). NcRNA regulated pyroptosis in liver diseases and traditional chinese medicine intervention: A narrative review.. J. Inflamm. Res..

[93] Shi C., Cao P., Wang Y., Zhang Q., Zhang D., Wang Y., Wang L., Gong Z. (2023). PANoptosis: A cell death characterized by pyroptosis, apoptosis, and necroptosis.. J. Inflamm. Res..

[94] Kong Q., Xia S., Pan X., Ye K., Li Z., Li H., Tang X., Sahni N., Yi S.S., Liu X., Wu H., Elowitz M.B., Lieberman J., Zhang Z. (2023). Alternative splicing of *GSDMB* modulates killer lymphocyte–triggered pyroptosis.. Sci. Immunol..

[95] Zhou Z., He H., Wang K., Shi X., Wang Y., Su Y., Wang Y., Li D., Liu W., Zhang Y., Shen L., Han W., Shen L., Ding J., Shao F. (2020). Granzyme A from cytotoxic lymphocytes cleaves GSDMB to trigger pyroptosis in target cells.. Science.

[96] LaRock D.L., Johnson A.F., Wilde S., Sands J.S., Monteiro M.P., LaRock C.N. (2022). Group A Streptococcus induces GSDMA-dependent pyroptosis in keratinocytes.. Nature.

[97] Wei Y., zhang Y., Zhan B., Wang Y., Cheng J., Yu H., Lv M., Zhang Y., Zhai Y., Guan Y., Feng H. (2025). Asiaticoside alleviated NAFLD by activating Nrf2 and inhibiting the NF-κB pathway.. Phytomedicine.

[98] Engin A. (2017). Non-alcoholic fatty liver disease.. Adv. Exp. Med. Biol..

[99] Xu W., Shao L., Zhou C., Wang H., Guo J. (2011). Upregulation of Nrf2 expression in non-alcoholic fatty liver and steatohepatitis.. Hepatogastroenterology.

[100] Seedorf K., Weber C., Vinson C., Berger S., Vuillard L.M., Kiss A., Creusot S., Broux O., Geant A., Ilic C., Lemaitre K., Richard J., Hammoutene A., Mahieux J., Martiny V., Durand D., Melchiore F., Nyerges M., Paradis V., Provost N., Duvivier V., Delerive P. (2023). Selective disruption of NRF2-KEAP1 interaction leads to NASH resolution and reduction of liver fibrosis in mice.. JHEP Rep. Innov. Hepatol..

[101] Bellezza I., Giambanco I., Minelli A., Donato R. (2018). Nrf2-Keap1 signaling in oxidative and reductive stress.. Biochim. Biophys. Acta Mol. Cell Res..

[102] Kakiyama G., Rodriguez-Agudo D., Pandak W.M. (2023). Mitochondrial cholesterol metabolites in a bile acid synthetic pathway drive nonalcoholic fatty liver disease: A revised “two-hit” hypothesis.. Cells.

[103] Tanase D.M., Gosav E.M., Costea C.F., Ciocoiu M., Lacatusu C.M., Maranduca M.A., Ouatu A., Floria M. (2020). The intricate relationship between type 2 diabetes mellitus (T2DM), insulin resistance (IR), and nonalcoholic fatty liver disease (NAFLD).. J. Diabetes Res..

[104] Kay H.Y., Kim W.D., Hwang S.J., Choi H.S., Gilroy R.K., Wan Y.J.Y., Kim S.G. (2011). Nrf2 inhibits LXRα-dependent hepatic lipogenesis by competing with FXR for acetylase binding.. Antioxid. Redox Signal.

[105] Shen B., Feng H., Cheng J., Li Z., Jin M., Zhao L., Wang Q., Qin H., Liu G. (2020). Geniposide alleviates non-alcohol fatty liver disease *via* regulating Nrf2/AMPK/mTOR signalling pathways.. J. Cell Mol. Med..

[106] Farzaei M.H., Zobeiri M., Parvizi F., El-Senduny F.F., Marmouzi I., Coy-Barrera E., Naseri R., Nabavi S.M., Rahimi R., Abdollahi M. (2018). Curcumin in liver diseases: A systematic review of the cellular mechanisms of oxidative stress and clinical perspective.. Nutrients.

[107] Feng X., Yu W., Li X., Zhou F., Zhang W., Shen Q., Li J., Zhang C., Shen P. (2017). Apigenin, a modulator of PPARγ, attenuates HFD-induced NAFLD by regulating hepatocyte lipid metabolism and oxidative stress *via* Nrf2 activation.. Biochem. Pharmacol..

[108] Xu D., Xu M., Jeong S., Qian Y., Wu H., Xia Q., Kong X. (2019). The role of Nrf2 in liver disease: Novel molecular mechanisms and therapeutic approaches.. Front Pharmacol..

[109] Ma Y., Lee G., Heo S.Y., Roh Y.S. (2021). Oxidative stress is a key modulator in the development of nonalcoholic fatty liver disease.. Antioxidants.

[110] Peverill W., Powell L., Skoien R. (2014). Evolving concepts in the pathogenesis of NASH: Beyond steatosis and inflammation.. Int. J. Mol. Sci..

[111] Gan L., Johnson J.A. (2014). Oxidative damage and the Nrf2-ARE pathway in neurodegenerative diseases.. Biochim. Biophys. Acta Mol. Basis Dis..

[112] Vasconcelos A.R., dos Santos N.B., Scavone C., Munhoz C.D. (2019). Nrf2/ARE pathway modulation by dietary energy regulation in neurological disorders.. Front Pharmacol..

[113] Ghanim B.Y., Qinna N.A. (2022). Nrf2/ARE axis signalling in hepatocyte cellular death.. Mol. Biol. Rep..

[114] Mohs A., Otto T., Schneider K.M., Peltzer M., Boekschoten M., Holland C.H., Hudert C.A., Kalveram L., Wiegand S., Saez-Rodriguez J., Longerich T., Hengstler J.G., Trautwein C. (2021). Hepatocyte-specific NRF2 activation controls fibrogenesis and carcinogenesis in steatohepatitis.. J. Hepatol..

[115] Liu B., Deng X., Jiang Q., Li G., Zhang J., Zhang N., Xin S., Xu K. (2020). Scoparone improves hepatic inflammation and autophagy in mice with nonalcoholic steatohepatitis by regulating the ROS/P38/Nrf2 axis and PI3K/AKT/mTOR pathway in macrophages.. Biomed. Pharmacother..

[116] Peng W., He C.X., Li R.L., Qian D., Wang L.Y., Chen W.W., Zhang Q., Wu C.J. (2024). Zanthoxylum bungeanum amides ameliorates nonalcoholic fatty liver *via* regulating gut microbiota and activating AMPK/Nrf2 signaling.. J. Ethnopharmacol..

[117] Wang X., Monemi M., Moghadam F.M., Fazilat A., Roshani S., Valilo M. (2025). The crosstalk between Nrf2 and pyroptosis in diseases.. Pathol. Res. Pract..

[118] Stępkowski T.M., Kruszewski M.K. (2011). Molecular cross-talk between the NRF2/KEAP1 signaling pathway, autophagy, and apoptosis.. Free Radic. Biol. Med..

[119] Greaney A.J., Maier N.K., Leppla S.H., Moayeri M. (2016). Sulforaphane inhibits multiple inflammasomes through an Nrf2-independent mechanism.. J. Leukoc. Biol..

[120] Alharbi K.S., Almalki W.H., Albratty M., Meraya A.M., Najmi A., Vyas G., Singh S.K., Dua K., Gupta G. (2022). The therapeutic role of nutraceuticals targeting the Nrf2/ HO -1 signaling pathway in liver cancer.. J. Food Biochem..

[121] Zhang Q., Liu J., Duan H., Li R., Peng W., Wu C. (2021). Activation of Nrf2/HO-1 signaling: An important molecular mechanism of herbal medicine in the treatment of atherosclerosis *via* the protection of vascular endothelial cells from oxidative stress.. J. Adv. Res..

[122] Zhang X., Yu Y., Lei H., Cai Y., Shen J., Zhu P., He Q., Zhao M. (2020). The Nrf-2/HO-1 signaling axis: A ray of hope in cardiovascular diseases.. Cardiol. Res. Pract..

[123] Yang W., Wang Y., Huang Y., Wang T., Li C., Zhang P., Liu W., Yin Y., Li R., Tao K. (2024). Immune Response Gene-1 [IRG1]/itaconate protect against multi-organ injury *via* inhibiting gasdermin D-mediated pyroptosis and inflammatory response.. Inflammopharmacology.

[124] Yang W., Wang Y., Zhang P., Sun X., Chen X., Yu J., Shi L., Yin Y., Tao K., Li R. (2022). Immune-responsive gene 1 protects against liver injury caused by concanavalin A *via* the activation Nrf2/HO-1 pathway and inhibition of ROS activation pathways.. Free Radic. Biol. Med..

[125] El-Gohary R.M., Ghalwash A.A., Awad M.M., El-Shaer R.A.A., Ibrahim S., Eltantawy A.F., Elmansy A., Okasha A.H. (2023). Novel insights into the augmented effect of curcumin and liraglutide in ameliorating cisplatin-induced nephrotoxicity in rats: Effects on oxidative stress, inflammation, apoptosis and pyroptosis *via* GSK-3β.. Arch. Biochem. Biophys..

[126] Wang L., Liu J., Wang Z., Qian X., Zhao Y., Wang Q., Dai N., Xie Y., Zeng W., Yang W., Bai X., Yang Y., Qian J. (2023). Dexmedetomidine abates myocardial ischemia reperfusion injury through inhibition of pyroptosis *via* regulation of miR-665/MEF2D/Nrf2 axis.. Biomed. Pharmacother..

[127] Long J.X., Tian M.Z., Chen X.Y., Yu H.H., Ding H., Liu F., Du K. (2023). The role of NLRP3 inflammasome-mediated pyroptosis in ischemic stroke and the intervention of traditional chinese medicine.. Front. Pharmacol..

[128] Zhang Q., Hu Y., Hu J.E., Zhang M. (2022). Solasonine alleviates high glucose-induced podocyte injury through increasing Nrf2-medicated inhibition of NLRP3 activation.. Drug Dev. Res..

[129] Zhaolin Z., Guohua L., Shiyuan W., Zuo W. (2019). Role of pyroptosis in cardiovascular disease.. Cell Prolif..

[130] Pang Y., Zhang P., Lu R., Li H., Li J., Fu H., Cao Y.W., Fang G., Liu B., Wu J., Zhou J., Zhou Y. (2020). Andrade-oliveira salvianolic Acid B modulates caspase-1–mediated pyroptosis in renal ischemia-reperfusion injury *via* Nrf2 pathway.. Front. Pharmacol..

[131] Wang J., Ye W., Zou J., Yang P., Jin M., Zheng Z., Zhou C., Qiu W., Lu J., Li C., Guo S., Xu Y., Huang Z., Liu P., Liu Z. (2024). Targeting the smooth muscle cell Keap1-Nrf2-GSDMD-pyroptosis axis by cryptotanshinone prevents abdominal aortic aneurysm formation.. Theranostics.

[132] Lv Y., Gao X., Luo Y., Fan W., Shen T., Ding C., Yao M., Song S., Yan L. (2019). Apigenin ameliorates HFD-induced NAFLD through regulation of the XO/NLRP3 pathways.. J. Nutr. Biochem..

[133] Yang G., Lee H.E., Lee J.Y. (2016). A pharmacological inhibitor of NLRP3 inflammasome prevents non-alcoholic fatty liver disease in a mouse model induced by high fat diet.. Sci. Rep..

[134] Hu C., Zhao L., Tao J., Li L. (2019). Protective role of melatonin in early-stage and end-stage liver cirrhosis.. J. Cell Mol. Med..

[135] Ahmadi Z., Ashrafizadeh M. (2020). Melatonin as a potential modulator of Nrf2.. Fundam. Clin. Pharmacol..

[136] Cai J., Yang J., Chen X., Zhang H., Zhu Y., Liu Q., Zhang Z. (2022). Melatonin ameliorates trimethyltin chloride-induced cardiotoxicity: The role of nuclear xenobiotic metabolism and Keap1-Nrf2 / ARE axis-mediated pyroptosis.. Biofactors.

[137] Yen I.C., Tu Q.W., Chang T.C., Lin P.H., Li Y.F., Lee S.Y. (2021). 4-Acetylantroquinonol B ameliorates nonalcoholic steatohepatitis by suppression of ER stress and NLRP3 inflammasome activation.. Biomed. Pharmacother..

[138] Shi H., Zhang Y., Xing J., Liu L., Qiao F., Li J., Chen Y. (2020). Baicalin attenuates hepatic injury in non-alcoholic steatohepatitis cell model by suppressing inflammasome-dependent GSDMD-mediated cell pyroptosis.. Int. Immunopharmacol..

[139] Shi H., Qiao F., Lu W., Huang K., Wen Y., Ye L., Chen Y. (2022). Baicalin improved hepatic injury of NASH by regulating NRF2/HO-1/NRLP3 pathway.. Eur. J. Pharmacol..

[140] Kuo N.C., Huang S.Y., Yang C.Y., Shen H.H., Lee Y.M. (2020). Involvement of HO-1 and autophagy in the protective effect of magnolol in hepatic steatosis-induced NLRP3 inflammasome activation *in vivo and in vitro.*. Antioxidants.

[141] Hwangbo H., Kim M.Y., Ji S.Y., Kim S.Y., Lee H., Kim G.Y., Park C., Keum Y.S., Hong S.H., Cheong J., Choi Y.H. (2020). Auranofin attenuates non-alcoholic fatty liver disease by suppressing lipid accumulation and NLRP3 inflammasome-mediated hepatic inflammation *in vivo and in vitro.*. Antioxidants.

[142] Chen H.W., Yen C.C., Kuo L.L., Lo C.W., Huang C.S., Chen C.C., Lii C.K. (2020). Benzyl isothiocyanate ameliorates high-fat/cholesterol/cholic acid diet-induced nonalcoholic steatohepatitis through inhibiting cholesterol crystal-activated NLRP3 inflammasome in Kupffer cells.. Toxicol. Appl. Pharmacol..

[143] Lo C.W., Yen C.C., Chen C.Y., Chen H.W., Lii C.K. (2023). Benzyl isothiocyanate attenuates activation of the NLRP3 inflammasome in Kupffer cells and improves diet-induced steatohepatitis.. Toxicol. Appl. Pharmacol..

[144] Mai W., Xu Y., Xu J., Zhao D., Ye L., Yu G., Wang Z., Lu Q., Lin J., Yang T., Gu C., Liu S., Zhong Y., Yang H. (2020). Berberine inhibits nod-like receptor family pyrin domain containing 3 inflammasome activation and pyroptosis in nonalcoholic steatohepatitis *via* the ROS/TXNIP Axis.. Front. Pharmacol..

[145] Ding B., Geng S., Hou X., Ma X., Xu H., Yang F., Liu K., Liang W., Ma G. (2021). Berberine reduces renal cell pyroptosis in golden hamsters with diabetic nephropathy through the Nrf2-NLRP3-Caspase-1-GSDMD pathway.. Evid. Based Complement. Alternat. Med..

[146] Wang Y., Liu F., Liu M., Zhou X., Wang M., Cao K., Jin S., Shan A., Feng X. (2022). Curcumin mitigates aflatoxin B1-induced liver injury *via* regulating the NLRP3 inflammasome and Nrf2 signaling pathway.. Food Chem. Toxicol..

[147] Wei Z., pinfang K., jing Z., zhuoya Y., Shaohuan Q., Chao S. (2023). Curcumin improves diabetic cardiomyopathy by inhibiting pyroptosis through AKT/Nrf2/ARE pathway.. Mediators Inflamm..

[148] Biao Y., Chen J., Liu C., Wang R., Han X., Li L., Zhang Y. (2022). Protective effect of *danshen zexie* decoction against non-alcoholic fatty liver disease through inhibition of ROS/NLRP3/IL-1β pathway by Nrf2 signaling activation.. Front. Pharmacol..

[149] Shi M., Huang F., Deng C., Wang Y., Kai G. (2019). Bioactivities, biosynthesis and biotechnological production of phenolic acids in *Salvia miltiorrhiza*.. Crit. Rev. Food Sci. Nutr..

[150] Shao N., Yu X.Y., Ma X.F., Lin W.J., Hao M., Kuang H.Y. (2018). Exenatide delays the progression of nonalcoholic fatty liver disease in C57BL/6 mice, which may involve inhibition of the NLRP3 inflammasome through the mitophagy pathway.. Gastroenterol. Res. Pract..

[151] Wang F., Park J.S., Ma Y., Ma H., Lee Y.J., Lee G.R., Yoo H.S., Hong J.T., Roh Y.S. (2021). Ginseng saponin enriched in Rh1 and Rg2 ameliorates nonalcoholic fatty liver disease by inhibiting inflammasome activation.. Nutrients.

[152] Shen B., Wang Y., Cheng J., Peng Y., Zhang Q., Li Z., Zhao L., Deng X., Feng H. (2023). Pterostilbene alleviated NAFLD *via* AMPK/mTOR signaling pathways and autophagy by promoting Nrf2.. Phytomedicine.

[153] Naderi M., Salavatiha Z., Gogoi U., Mohebbi A. (2024). An overview of anti-Hepatitis B virus flavonoids and their mechanisms of action.. Front Cell Infect. Microbiol..

[154] Cox A.R., Masschelin P.M., Saha P.K., Felix J.B., Sharp R., Lian Z., Xia Y., Chernis N., Bader D.A., Kim K.H., Li X., Yoshino J., Li X., Li G., Sun Z., Wu H., Coarfa C., Moore D.D., Klein S., Sun K., Hartig S.M. (2022). The rheumatoid arthritis drug auranofin lowers leptin levels and exerts antidiabetic effects in obese mice.. Cell Metab..

[155] Lin X., Zhang J., Chu Y., Nie Q., Zhang J. (2024). Berberine prevents NAFLD and HCC by modulating metabolic disorders.. Pharmacol. Ther..

[156] Li R., Li X., Zhao J., Meng F., Yao C., Bao E., Sun N., Chen X., Cheng W., Hua H., Li X., Wang B., Wang H., Pan X., You H., Yang J., Ikezoe T. (2022). Mitochondrial STAT3 exacerbates LPS-induced sepsis by driving CPT1a-mediated fatty acid oxidation.. Theranostics.

[157] Feng Y., Chen Y., Yang B., Lan Q., Wang T., Cui G., Ren Z., Choi I.C., Leung G.P.H., Yan F., Chen D., Yu H.H., Lee S.M.Y. (2019). Hepatoprotective effect of jianpi huoxue formula on nonalcoholic fatty liver disease induced by methionine-choline-deficient diet in rat.. BioMed. Res. Int..

[158] Arzimanoglou A., Brandl U., Cross J.H., Gil-Nagel A., Lagae L., Landmark C.J., Specchio N., Nabbout R., Thiele E.A., Gubbay O. (2020). Epilepsy and cannabidiol: A guide to treatment.. Epileptic. Disord..

[159] Mantovani A., Petracca G., Beatrice G., Csermely A., Lonardo A., Targher G. (2021). Glucagon-Like Peptide-1 receptor agonists for treatment of nonalcoholic fatty liver disease and nonalcoholic steatohepatitis: An updated meta-analysis of randomized controlled trials.. Metabolites.

[160] Nauck M.A., Quast D.R., Wefers J., Meier J.J. (2021). GLP-1 receptor agonists in the treatment of type 2 diabetes – state-of-the-art.. Mol. Metab..

[161] Xu C., Song Y., Wang Z., Jiang J., Piao Y., Li L., Jin S., Li L., Zhu L., Yan G. (2021). Pterostilbene suppresses oxidative stress and allergic airway inflammation through AMPK/Sirt1 and Nrf2/HO-1 pathways.. Immun. Inflamm. Dis..

